# Weight Change in a Commercial Web-Based Weight Loss Program and its Association With Website Use: Cohort Study

**DOI:** 10.2196/jmir.1756

**Published:** 2011-10-12

**Authors:** Melinda Neve, Philip J Morgan, Clare E Collins

**Affiliations:** ^1^School of Health SciencesFaculty of HealthUniversity of NewcastleCallaghan, NSWAustralia; ^2^School of EducationFaculty of Education and ArtsUniversity of NewcastleCallaghanAustralia; ^3^Priority Research Centre in Physical Activity and NutritionUniversity of NewcastleCallaghan, NSWAustralia

**Keywords:** Weight loss, obesity, intervention, Internet, commercial

## Abstract

**Background:**

There is a paucity of information in the scientific literature on the effectiveness of commercial weight loss programs, including Web-based programs. The potential of Web-based weight loss programs has been acknowledged, but their ability to achieve significant weight loss has not been proven.

**Objective:**

The objectives were to evaluate the weight change achieved within a large cohort of individuals enrolled in a commercial Web-based weight loss program for 12 or 52 weeks and to describe participants’ program use in relation to weight change.

**Method:**

Participants enrolled in an Australian commercial Web-based weight loss program from August 15, 2007, through May 31, 2008. Self-reported weekly weight records were used to determine weight change after 12- and 52-week subscriptions. The primary analysis estimated weight change using generalized linear mixed models (GLMMs) for all participants who subscribed for 12 weeks and also for those who subscribed for 52 weeks. A sensitivity analysis was conducted using the last observation carried forward (LOCF) method. Website use (ie, the number of days participants logged on, made food or exercise entries to the Web-based diary, or posted to the discussion forum) was described from program enrollment to 12 and 52 weeks, and differences in website use by percentage weight change category were tested using Kruskal-Wallis test for equality of populations.

**Results:**

Participants (n = 9599) had a mean (standard deviation [SD]) age of 35.7 (9.5) years and were predominantly female (86% or 8279/9599) and obese (61% or 5866/9599). Results from the primary GLMM analysis including all enrollees found the mean percentage weight change was −6.2% among 12-week subscribers (n = 6943) and −6.9% among 52-week subscribers (n = 2656). Sensitivity analysis using LOCF revealed an average weight change of −3.0% and −3.5% after 12 and 52 weeks respectively. The use of all website features increased significantly (*P* < .01) as percentage weight change improved.

**Conclusions:**

The weight loss achieved by 12- and 52-week subscribers of a commercial Web-based weight loss program is likely to be in the range of the primary and sensitivity analysis results. While this suggests that, on average, clinically important weight loss may be achieved, further research is required to evaluate the efficacy of this commercial Web-based weight loss program prospectively using objective measures. The potential association between greater website use and increased weight loss also requires further evaluation, as strategies to improve participants’ use of Web-based program features may be required.

## Introduction

As the prevalence of overweight and obesity among adults continues to increase across the world [1], the need for cost-effective programs that achieve clinically important weight loss and have a broad reach are urgently needed. However, at this time there is no universally effective method of weight management that assures long-term maintenance of lost weight [2,3]. Despite this, many overweight and obese men (44%) and women (65%) report trying to lose weight [4], and many of these individuals join commercial weight loss programs [5].

The most recent systematic review of major commercial weight loss programs concluded that there was inadequate evidence to recommend their use [5], and that further randomized controlled trials (RCT) were required to provide evidence to support or refute the use of commercial weight loss programs [5]. Although controlled trials are essential to demonstrate efficacy, the results of these trials may not always be generalizable to typical enrollees of commercial programs. Therefore, studies evaluating outcomes for fee-paying commercial weight loss program participants can establish the nature of consumer engagement and the degree of weight loss that can be expected after specific periods of enrollment [6].

Commercial weight loss program providers commonly offer Web-based versions of their programs. Recent systematic reviews of Web-based weight loss interventions have highlighted the potential of these programs to achieve significant weight loss [7-10]. However, only one commercial Web-based weight loss program (ie, eDiets) has undergone rigorous testing within two RCTs, conducted in 2004 and 2007 [11,12]. The first RCT found that after 12 months, participants in the program achieved significantly less weight loss compared with participants given a self-help manual, [11]. The second RCT compared eDiets with a structured behavioral Web-based program and found that participants in the behavioral program achieved significantly greater weight loss compared with those participants randomized to eDiets after 12 months [12]. Therefore, further research is required to evaluate the weight change achieved from participation in commercial Web-based weight loss programs.

A 2010 systematic review of Web-based weight loss interventions found that greater weight loss is likely to be associated with increased use of Web-based program features [9]. This is consistent with the results of the majority of studies investigating the association between intervention exposure and outcomes, that is, greater use of program components within Web-based interventions is associated with greater weight loss or better weight loss maintenance. Program components include log-ins [12-20], self-monitoring of weight, diet and/or exercise [11,12,18,20-25], attendance at online meetings or chat sessions [12,22,25], forum posts [12,22], viewing online lessons [21], as well as overall website use [26]. Therefore, a vital component of achieving successful weight outcomes through Web-based weight loss interventions appears to be their ability to engage participants. However, we have limited knowledge of whether the association between website use and weight loss holds true for fee-paying members of commercial Web-based programs.

Therefore, the primary aim of this study was to describe the weight loss achieved by a cohort of enrollees of a commercial Web-based weight loss program among participants who subscribed to the program for 12 or 52 weeks. The secondary aim was to describe participants’ use of the Web-based program overall and by percentage weight loss category and to determine if website use differed by percentage weight loss category.

## Methods

### Participants and Design

Participants were eligible for inclusion in the study if they paid for a subscription to the program from August 15, 2007, through May 31, 2008. To join the program, participants must have been 18 to 75 years of age and have had a body mass index (BMI) greater than or equal to 22 kg/m^2^ based on self-reported height and weight. When participants enrolled, they purchased a subscription plan of 4-, 12-, 16- or 52-weeks duration. In 2007-2008, a subscription cost A$16.50 to A$79.95 per month dependant on the number of months a participant subscribed. Participants could not unsubscribe from their selected plan until the subscription timeframe had elapsed unless they had special circumstances that prevented them from completing their subscription (eg, pregnancy or financial difficulties). This study included participants who subscribed for the most popular durations of 12- or 52-weeks. Data related to free or non-consecutive subscriptions (≥ 7 days apart) were also excluded.

Characteristics of the full cohort [27] and the subgroup who subscribed for periods of 12 and 52 weeks [28] have been previously published.

### The Commercial Web-Based Weight Loss Program

In 2007-2008, SP Health Co Pty Ltd (Sydney, Australia) offered a Web-based weight loss platform that was commercially available in Australia as The Biggest Loser Club (www.biggestloserclub.com.au). It was promoted as a 12-week program, but participants could choose to subscribe for longer to assist with further weight loss and/or maintenance. The self-directed program incorporated evidence-based weight management strategies and aligned with key elements of social cognitive theory [29]. Participants set a goal weight and were encouraged to work towards this target in “mini goals” (eg, 5kg or 5%). Participants were encouraged to self-monitor by reporting their weight or other body measurements via the website or short message service (SMS) and could view graphs and charts detailing their progress over time (eg, weight and waist circumference change). Participants were encouraged to weigh in once per week and received weekly reminders to do so via email or SMS during the initial 12-week program. A daily energy intake target was set based on the participant’s sex, weight, height, and physical activity level to facilitate either a weight loss of 0.5 kg to 1 kg per week or maintain current weight. Participants were encouraged to self-monitor their dietary intake and exercise using an online diary that calculated daily energy intake and expenditure. Online information in the form of weekly tutorials, fact sheets, meal, and exercise plans and weekly challenges were provided during the initial 12-week program. After 12-weeks, participants continued to receive weekly Web-based tutorials. Participants were also prompted to access the online information via a weekly email. Social support was available via a discussion board to communicate with other members.

### Data Collection

All data were collected by SP Health Co, provided to the researcher in deidentified form, and included enrollment survey responses (anthropometric measures, ie, weight and height, and demographics, ie, age, gender, and postcode), subscription data (date of enrollment, date membership ceased, and subscription plans held), website use (date of log-in, online food and exercise diary entries, and posts to the discussion forum), and self-reported weight records (date of record and weight recorded). Ethics approval for the study was obtained from the University of Newcastle Human Research Ethics Committee. 

### Measures

Participants’ characteristics were captured from the enrollment survey. Self-reported height and weight were used to calculate BMI (weight in kg/height in m^2^), which was categorized as healthy, overweight, or obese using the World Health Organization’s (WHO) BMI classification [30]. Reported postcodes were assigned an Index of Relative Socioeconomic Advantage and Disadvantage (IRSAD) tertile (ranked from 1 = disadvantage to 10 = advantaged) [31] as an indicator of socioeconomic status, as well as an Accessibility/Remoteness Index of Australia (ARIA) [32] to classify residential level of remoteness.

Data relating to the subscription plans participants held were used to determine whether participants enrolled for 12 or 52 weeks. The date of enrollment and the date that membership ceased were used to calculate the number of days each participant was a member of the program and, therefore, how many participants cancelled their subscription. The self-reported weight records were used to describe the number of people who weighed in each week. The self-reported weights (in kilograms) were used to determine the weight change achieved. The total number of days per week each of the website features (log-ins, food diary entries, exercise diary entries, and forum posts) were used was calculated to describe overall website use.

### Data Analysis

Data analysis was undertaken using Stata 11.0 (StataCorp, College Station, Texas, USA), with *P* values less than .01 considered statistically significant. Descriptive statistics are described as means and standard deviations (SDs) for normally distributed continuous variables, medians and interquartile ranges (IQR) for nonnormal continuous data, and percentages for categorical variables.

Absolute and percentage weight change were calculated from enrollment to 12 weeks for participants who subscribed for 12 weeks and from enrollment to 52 weeks for participants who subscribed for 52 weeks. The primary analysis, to determine the weight change achieved by all program enrollees, was conducted using generalized linear mixed models (GLMMs) containing available self-reported weight records for all participants. GLMM was used because this is the preferred method for longitudinal data with missing values [33,34]. Baseline age, BMI, socioeconomic status, and remoteness were controlled for in the analyses as potential confounders.

A secondary sensitivity analysis was conducted to determine the robustness of the results from the GLMM approach. This analysis was required as GLMM are based on the assumption that missing data are missing at random, which many not be the case for data reported as part of a weight loss program. Therefore, a sensitivity analysis was conducted by imputing missing data for weight using the last observation carried forward (LOCF) method.

Spearman’s rank correlations were calculated to explore associations of weight change with website use. This included the percentage weight change results from the LOCF analyses. Participants were divided into four percentage weight loss categories (weight gain, 0% to < 5% weight loss, 5% to < 10% weight loss, and ≥ 10% weight loss) based on the LOCF analysis results. The median and IQR website use was described by percentage weight loss group and differences between groups investigated using Kruskal-Wallis test for equality of populations.

## Results

### Participant Characteristics

**Figure 1:**
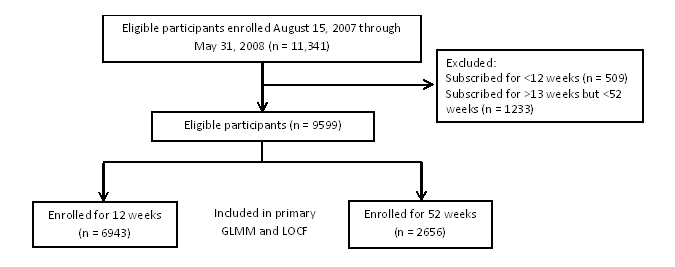
Participant flow through the trial.

Participant flow is reported in Figure 1. A total of 11,341 participants subscribed to the program from August 15, 2007, through May 31, 2008. This study included 9599 participants; 6943 subscribed to the program for 12 weeks and 2656 subscribed for 52 weeks. Participant characteristics at enrollment have been described in detail elsewhere [27]. In summary, participants had a mean (SD) age of 35.7 (9.5) years and were predominantly female (86% or 8279/9599), obese (61% or 5866/9599), of moderate to high socioeconomic status (85% or 8022/9455 scored between 5 and 10 on IRSAD), and from major cities in Australia (75% or 7125/9456). Participants who subscribed for 12 weeks were significantly younger (35.3 years of age vs 36.7 years of age), had lower BMI (31.8 vs 35.8), were of higher socioeconomic status (39.1% vs 32.8% IRSAD 9-10), and were more likely to live in major cities of Australia (76.4% vs 72.7%), compared with 52-week subscribers. In addition, 3% (238/6943) of 12-week subscribers and 23% (605/2656) of 52-week subscribers cancelled their subscription during their subscription period due to special circumstances.

### Self-Reported Weight Record

**Figure 2:**
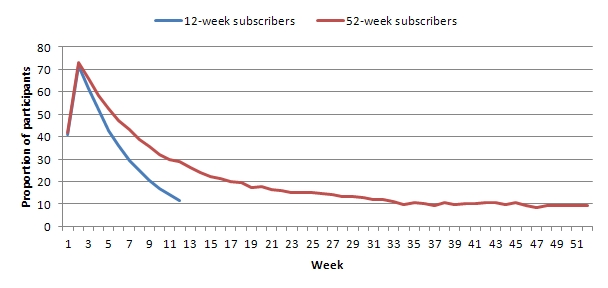
Percent of participants who weighed in per week for 12- and 52-week subscribers.

The proportion of participants who self-reported their weight each week declined substantially over time (Figure 2). Therefore, the amount of missing weight data increased. For both 12- and 52-week subscribers, the highest proportion of participants self-reported their weight during week 2 (72% and 73%). For 12-week subscribers, only 11% (792/6943) self-reported their weight during their final week of the program (ie, 89% of participants’ weight data was missing). For 52-week subscribers, the decline in the number of participants self-reporting their weight was continuous from week 2 (73%) to week 32 (12%). However, after week 32, the percentage of participants self-reporting a weight reached a plateau but remained steady at 9% to 11% until 52 weeks. Therefore, 91% (2412 /2656) of participants’ weight data was missing at week 52.

### Weight Change: Primary Analysis

Weight change results for 12- and 52-week subscribers are shown in Table 1. The GLMM gave a mean self-reported weight reduction for 12-week subscribers of −5.6 kg (95% confidence interval [CI] −5.8 kg to −5.5 kg) or −6.2% and included an average of 5.2 weekly self-reported weight records per participant. The mean self-reported weight change among 52-week subscribers was −8.4 kg (95% CI −9.0 kg to −7.8 kg) or −6.9% from the GLMM. The analysis included an average of 11.8 weekly self-reported weight records per participant.

### Weight Change: Sensitivity Analysis

The sensitivity analysis using LOCF gave a mean self-reported weight loss of −2.6 kg (95% CI −2.7 kg to −2.5 kg) or −3.0%, and 21% (1479/6943) achieved greater than or equal to 5% weight loss after 12 weeks (Table 1). The sensitivity analysis using LOCF gave a mean self-reported weight change of −3.6 kg (95% CI −3.8 kg to −3.3 kg) or −3.5% from baseline to 52 weeks with 29% (777/2656) of participants achieving greater than or equal to 5% weight loss from enrollment to 52 weeks (Table 1).

**Table 1:** Mean (95% CI) weight change for a cohort of participants who subscribed to a commercial Web-based weight loss program for 12 or 52 weeks using GLMM and LOCF analyses

Cohort and Weight Change Measure			GLMM Analysis^a,b^	LOCF Analysis^a,b^
**12-week subscribers (n = 6943)**				
	Absolute weight change (95% CI)		−5.6 kg (−5.8 kg to −5.5 kg)	−2.6 kg (−2.7 kg to −2.5 kg)
	Percentage weight change (95% CI)		−6.2% (−6.3% to −6.1%)	−3.0% (−3.0% to −2.9%)
	**Percentage weight change category**			
		Weight gain, n (%)		423 (6.1%)
		0% to < 5%, n (%)		5041 (72.6%)
		5% to < 10%, n (%)		1206 (17.4%)
		10% or more, n (%)		273 (3.9%)
**52-week subscribers (n = 2656)**				
	Absolute weight change (95% CI)		−8.4 kg (−9.0 kg to −7.8 kg)	−3.6 kg (−3.8 kg to −3.3 kg)
	Percentage weight change (95% CI)		−6.9% (−7.3% to −6.5%)	−3.5% (−3.8% to −3.3%)
	**Percentage weight change category**			
		Weight gain, n (%)		424 (16.0%)
		0% to < 5%, n (%)		1455 (54.8%)
		5% to < 10%, n (%)		475 (17.9%)
		10% or more, n (%)		302 (11.4%)

^a^Difference from baseline to 12 and 52 weeks is statistically significant for all analyses (*P* < .001).

^b^Controlled for baseline age, BMI, socioeconomic status, and remoteness

### Website Use

Website use for 12- and 52-week subscribers is presented in Table 2. To summarize, 12-week subscribers logged on to the website a median of 13 days. They made food entries to the Web-based diary a median of 7 days and exercise entries, a median of 3 days. The median number of days that 12-week subscribers posted to the discussion forum was zero. Among 52-week subscribers, the median number of days participants logged on was 21 days. They used the Web-based diary for food entries a median of 8 days and exercise entries a median 3 days, with a median of zero posts to the discussion forums. 

**Table 2:** Description of 12- and 52-week subscribers’ use of the website features

	12-Week Subscribers (n = 6943)		52-Week Subscribers (n = 2656)	
	Participants Who Used the Feature, n (%)	Median (IQR)	Participants Who Used the Feature, n (%)	Median (IQR)
Log-ins	6682 (96.2%)	13 (6-26)	2576 (97.0%)	21 (7-56)
Food diary entries	5244 (75.5%)	7 (1-20)	1993 (75.0%)	8 (1-34)
Exercise diary entries	4686 (67.5%)	3 (0-9)	1801 (67.8%)	3 (0-15)
Posts to the discussion forum	860 (12.4%)	0 (0-0)	1055 (39.7%)	0 (0-0)

### Website Use and Weight Change

For both 12- and 52-week subscribers, percentage weight change was significantly positively correlated (P < .001) with the number of days each website feature was used (Table 3). The strongest correlations were found between the number of days participants logged on and weight change for 12- and 52-week subscribers. The weakest correlations were found between forum posts and weight change in both subscription groups. The strongest correlations were in the 12-week subscription group for all website features except forum posts, where the correlation between forum posts and weight change was stronger among 52-week subscribers.

**Table 3:** Spearman correlations between website use and percentage weight change (kg) among 12- and 52-week subscribers

	12-Week Subscribers (n = 6943) r^a^	52-Week Subscribers (n = 2656) r^a^
Log-ins	−0.55	−0.43
Food diary entries	−0.39	−0.33
Exercise diary entries	−0.38	−0.33
Forum posts	−0.12	−0.18

^a^All are statistically significant (*P* < .001)

**Figure 3:**
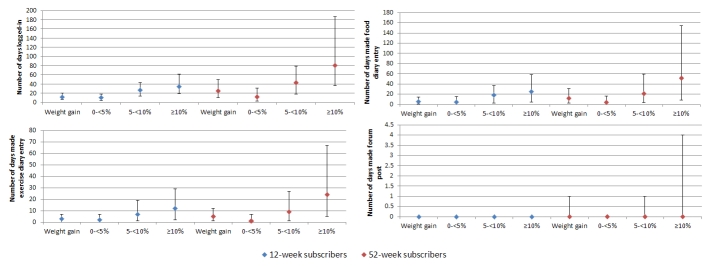
Median (IQR) days each website feature was used by 12- and 52-week subscribers by categories of percentage weight change.

The median number of days participants used each website feature increased significantly (*P* < .001) by category of higher percentage weight loss (Figure 3) for both 12- and 52-week subscribers. Among 12-week subscribers, those who lost 10% or more of their enrollment weight logged on a median of 34 days, made food entries to the Web-based diary 25 days, and made exercise entries 12 days, whereas those who gained weight logged on a median of 12 days, made food entries to the Web-based diary 6 days, and made exercise entries 3 days. For 52-week subscribers, those who lost 10% or more of enrollment weight logged on a median of 81 days, made food entries to the Web-based diary 52 days, and made exercise entries 24 days compared with those who gained weight, who had a median of 25 log-in days, used the Web-based food diary for food entries a median of 12 days, and made exercise entries for 5 days.

## Discussion

The primary aim of this paper was to describe the weight loss achieved by a large cohort of participants who subscribed to a commercial Web-based weight loss program for either 12 or 52 weeks. The study addresses an existing gap in the literature [5,6] by reporting weight loss outcomes in a large naturalistic cohort of commercial users of a Web-based weight loss program and its association with website feature usage. This study is one of only a small number of evaluations of commercial weight loss program cohorts and only the second to employ a robust statistical analysis as opposed to reporting results for program completers only. To the authors’ knowledge, it is the first cohort study reporting outcomes from a large group of enrollees in a commercial Web-based program.

### Weight Loss

Our primary analysis using GLMM indicated that both 12- and 52-week subscribers achieved statistically significant weight loss. Mean weight loss also exceeded the benchmark (≥ 5%) for clinically important weight loss and improvement in weight-related morbidity, particularly incidence of type 2 diabetes mellitus [35,36]. Furthermore, 21% of 12-week subscribers and 29% of 52-week subscribers achieved a weight loss greater than or equal to 5%, based on the results from the LOCF analysis.

However, the sensitivity analysis at both time points demonstrated less weight loss compared with the GLMM. GLMM assumes that any data missing from the model follow the same trajectory as the included data (in this case weekly weight change). As the average number of weekly weight records included was low and most people self-reported their weekly weight within the initial weeks of the program only, the GLMM results may be biased toward those who self-reported more weekly weights. It is likely that the participants who did not enter their weights were the less successful participants. This is supported by our previous findings that participants with poor eating and activity habits were more likely to stop using the program [28]. Furthermore, it is also likely that the rate of weight loss during the initial weeks of the program was higher compared with the later stages of the program; therefore, the trajectory of the GLMM may also be biased toward higher self-reported weight loss. Therefore, the true weight loss achieved by all participants at each time point is likely to be somewhere in the range between the GLMM and LOCF results (ie, −3.0% to −6.2% at 12 weeks and −3.5% to −6.9% at 52 weeks). Therefore, further research is required to confirm or refute these findings prospectively and objectively in a clinical research trial.

Results from the only two RCTs conducted using another commercial Web-based weight loss program, eDiets, reported a mean percentage weight change of −2.8% [12] and −1.1% [11] after 12 months. Both eDiets and the commercial Web-based program evaluated in this study included many of the components that have been suggested as key elements of successful Web-based weight management programs [37,38], such as self-monitoring, feedback, and social support. However, eDiets also included additional features not available in the program evaluated in the current study, such as online meetings, peer-mentoring [11,12], and face-to-face sessions with a psychologist [11]. It was expected that these additional program components would lead to greater weight change. However, the mean weight change achieved in the current study was greater. This is potentially due to the increased capabilities of the Internet since the first study was conducted and/or differences in study design. So, although both programs provided similar features, those in the current study may potentially have been more engaging, easier, and/or faster for participants to use, reducing the burden to adhere.

### Website Use and Weight Loss

The second aim of the paper was to describe participants’ use of the Web-based program and its features and to determine if website use was associated with degree of weight loss.

The study demonstrated a significant positive correlation between the number of times each website feature was used and weight change. Therefore, the results support previous research [9] suggesting that ongoing engagement with Web-based weight loss programs may enhance weight loss in the long-term. Given this association, strategies are required to encourage participants to use Web-based weight loss programs consistently to ensure that the majority of participants are given the opportunity to achieve clinically important weight loss.

However, at the group level, the average use of the commercial Web-based weight management program features appears to be low and inconsistent. The majority of subscribers log on and try the Web-based diary at least once; however, engagement decreases quite fast. This is demonstrated by the initial decline in weekly self-reported weight records over time for both 12- and 52-week subscribers and is consistent with other public health interventions delivered via the Internet, where usage declines after the initial weeks of the intervention [39].

As this commercial Web-based weight loss program is self-directed, the intensity or frequency of website use is not prescribed. Therefore, this study provides valuable data and insight into what level of website use may be feasible and, more importantly, what level is required to be effective in achieving weight change in a commercial setting. Interestingly, participants who achieved significant weight loss did not use the website unrealistically or excessively. For example, those who achieved greater than or equal to 10% weight loss from baseline to 12 weeks logged on approximately 40% of the possible days (34 days out of 84) and used the Web-based diary 30% of possible days (25 days out of 84). These findings suggest that developing program targets for weekly or monthly website use and for specific program features may increase usage and enhance weight loss, thus facilitating achievement of participants’ weight loss goals. However, to identify optimal exposure to the website overall, as well as individual website features, further investigation of the differences in use at different stages of the program and its association with weight loss is required. For example, this study demonstrates that participants who achieved greater than or equal to 10% weight loss from baseline to 12 weeks logged on approximately 40% of the possible days (34 days out of 84), whereas those who achieved the same percentage weight change from baseline to 52 weeks logged on approximately 22% of the days (81/365). Therefore, further research is needed to investigate the relationship between patterns of website use over time and the weight loss achieved at different time points.

### Limitations

There are several important considerations when interpreting the weight change results. First, the weight change results are based on self-report, and weight is commonly underreported [40]. However, self-reported weight recorded by participants of a Web-based weight loss program has been found to be accurate compared with measured weight [41]. Second, a notable number of weekly weight records were missing, as the weight data was entered voluntarily by participants as part of their program participation and many participants failed to do this. To address this, statistical analyses were conducted using GLMM. GLMMs are among the most robust statistical methods available as these models are less influenced by the bias introduced because of missing data. Additionally, a large number of individual weekly weight records were included in each analysis (31,228 and 36,339) allowing the analyses to be strongly powered. Therefore, the results from the statistical analysis provide us with an indication of the weight loss achieved by a cohort of enrollees of a commercial Web-based weight loss program. However, due to the low level of website use and, therefore, the very small number of participants still self-reporting their weight at the end of their subscription period, further research is required to confirm or refute these findings and to identify ways to increase participant engagement with the program

The website use data and the reported associations with weight change also have some limitations to be noted. First, the study did not consider use of all website features as these data were not available at the time of the study. Additional data concerning the use of all features (eg, weekly tutorials), as well as more detailed data on the reported features (eg, whether participants read the forum posts) would help to better understand participants’ engagement with the website and the relationship between weight loss and website use. Second, the analysis to determine if greater website use was associated with enhanced weight loss relied on the results of the LOCF analysis. As previously stated, the true weight loss achieved by all participants is likely to be somewhere in the range between the GLMM and LOCF results. Third, although an association between website use and weight loss was demonstrated, a large number of other factors may have influenced participants’ website use and/or weight loss (eg, self motivation, intention to change, and other weight loss strategies) that were not evaluated in this study. Therefore, the association between website use and weight loss must also be confirmed prospectively in an objective manner.

### Conclusion

In summary, this research provides important data on an underevaluated weight loss program medium in a large number of commercial program users. The weight loss achieved by 12- and 52-week subscribers of a commercial Web-based weight loss program is likely to be in the range of the primary and sensitivity analysis results. This suggests that, on average, clinically important weight loss may be achieved. The findings support the need for further research to evaluate the efficacy of Web-based weight loss programs and to assist in the development of strategies to increase participants’ ongoing use of Web-based program features.

## Abbreviations

ARIA: Accessibility/Remoteness Index of Australia
BMI: body mass index
CI: confidence interval
GLMM: generalized linear mixed model
ISRAD: Index of Relative Socioeconomic Advantage and Disadvantage
IQR: interquartile range
LOCF: last observation carried forward
